# Application of SolidWorks software in preoperative planning of high tibial osteotomy

**DOI:** 10.3389/fsurg.2022.951820

**Published:** 2023-01-06

**Authors:** Yufeng Lu, Xue Wang, Bo Yang, Zhaochen Xu, Baogang Zhang, Bin Jia, Jinlong He, Liang Qi, Min Wang, Feng Qiao

**Affiliations:** ^1^ Department of Integrated Traditional Chinese Medicine (TCM) and Western Medicine Orthopedics, Honghui Hospital, Xi'an Jiaotong University, Xi'an, China; ^2^ Department of Emergency Medicine, Affiliated Hospital of Shaanxi University of Traditional Chinese Medicine, Xianyang, China; ^3^ Graduate School, Xi'an Medical University, Xi'an, China

**Keywords:** open-wedge high tibial osteotomy, solidworks, preoperative planning, correction angle, weight-bearing line ratio

## Abstract

**Purpose:**

Open-wedge high tibial osteotomy (HTO) is a common surgical treatment for medial osteoarthritis in young and active patients. The accuracy of osteotomy is closely associated with postoperative efficacy. The accuracy of digital preoperative planning is higher than that of the preoperative manual measurement and several computer software with varying accuracy and convenience are used for digital preoperative planning. This study aimed to use the SolidWorks software for HTO preoperative planning and to determine its accuracy and reliability in HTO preoperative planning.

**Methods:**

We reviewed the data of 28 patients with 54 with medial compartment knee arthritis who underwent open-wedge HTO preoperative planning using SolidWorks between June 2019 and March 2021. The standard anteroposterior standing whole-leg radiographs were assessed before and 6 weeks after the surgery. The correction angle, weight-bearing line (WBL) ratio, mechanical femorotibial angle (mFTA), and medial proximal tibial angle (MPTA) before and after the surgery were compared. The clinical results were evaluated using the Knee Society score.

**Results:**

At 6 weeks after the surgery, the WBL ratio was corrected from 16.8% to 50.5%, mFTA was corrected from 6.4° varus to 1.2° valgus, and MPTA was corrected from 83.4° to 89.3°. No significant difference was observed between the predicted correction angle before the surgery and the correction angle measured 6 weeks after the surgery (*t* = −1.745, *p* = 0.087). The knee score and function score of Knee Society increased from 76.4 and 80.7 before surgery to 95.0 and 95.7, respectively.

**Conclusions:**

The SolidWorks software showed high accuracy and reliability in preoperative planning of open-wedge HTO in patients with medial compartment knee arthritis.

## Introduction

High tibial osteotomy (HTO) is an effective method for the treatment of medial single-compartment knee osteoarthritis as it corrects the weight-bearing line (WBL) of the lower limbs (1). Currently, lateral closing wedge HTO and open-wedge HTO (OWHTO) are most commonly used. Compared with lateral closing wedge HTO, OWHTO is less traumatic, simpler, more convenient for the intraoperative adjustment of lower limb alignment, more accurate in deformity correction, and easily convertible to total knee arthroplasty without the need for fibula osteotomy (2). OWHTO is widely used and has achieved a favorable outcome in young and active patients with medial compartment knee arthritis.

Previous studies have reported that the accuracy of limb alignment correction is important in determining the success of HTO. Both over-correction and under-correction can affect the clinical outcome and survival rate of patients with medial compartment knee arthritis after HTO. According to the biomechanical studies performed by Hsu et al. (3), the medial compartment of the knee joint with a 1.2° mechanical femorotibial angle (mFTA) varus deformity can share 75% of the weight in a single-leg weight-bearing. To improve the accuracy of OWHTO, designing the correction angle and the opening gap preoperatively is necessary to determine the target WBL passing through a certain point of the tibial plateau in standing whole-leg radiographs. Currently, the most commonly used preoperative planning methods are the Miniaci method (4–7), Dugdale–Noyes method (8, 9), and Coventry method (10). Studies have reported that the Miniaci method is reliable, convenient, and simple to measure the opening angle and gap. It is most commonly used for OWHTO preoperative planning. Because of the wide applications of the picture archiving and communication system (PACS), orthographic images can be magnified at desirable magnification using the computer, thus making radiographic measurement convenient in preoperative planning. Studies have reported that the use of PACS for preoperative planning is highly reliable (11–13). In recent years, several computer software has originated to assist surgeons in HTO preoperative planning, such as the Materialise OrthoView software, Osteotomy Master (14), Biomet Orthosize, mediCAD (15, 16), and PreOPlan (16), which can be used to import patients' full-length orthographic images of lower limbs to calculate the opening angle and gap of the osteotomy. The accuracy of osteotomy has been greatly improved using these techniques. Recently, computer navigation (17) and three-D printing individualized osteotomy templates (18) have been developed to improve the accuracy of osteotomy but they are expensive and cumbersome for preoperative planning.

Since 2019, researchers are using the SolidWorks software in our institution for OWHTO preoperative planning, and good results have been achieved. This study aimed to evaluate the accuracy and reliability of the SolidWorks software for preoperative planning in patients undergoing medial OWHTO by comparing the preoperative and postoperative WBL ratio and opening angle.

## Patients and methods

The study protocol was approved by the Institutional Review Board of our hospital (approval no. 202109009). Written informed consent was obtained from all participants.

We reviewed the medial open-wedge HTO performed by our center from June 2019 to April 2021.

The subject inclusion criteria were as follows: (1) age <45 years, (2) preoperative plan to use the SolidWorks software, (3) preoperative planning to design the knee joint weight line at 50% of the tibial plateau, and (4) knee varus deformity, varus <15°, knee joint Medial pain, osteoarthritis grade K-L 0-I grade; and (5) no restriction of the knee joint movement.

The study exclusion criteria were as follows: (1) presence of lesions in the lateral compartment of the knee joint; (2) knee joint ligament injuries, including medial and lateral laxity, instability of varus and valgus, and anterior and posterior cruciate ligament injuries; (3) obese patients, with BMI >30 kg/m^2^, and (4) inflammation arthritis such as rheumatoid arthritis.

Using these criteria, 54 knees of 28 patients (15 women and 13 men) were included. There were a total of 26 left knees and 28 right knees. Of the 26 bilateral HTOs, 25 were performed bilaterally at one stage. One case underwent a procedure on the right side first, followed by that on the left side after 5 months. The mean patient age at the time of index operation was 32.6 ± 7.7 years (range: 18–44 years). The mean follow-up was 25.6 ± 6.8 months (range: 4–26 months) (Table 1).

**Table 1:** Patient demographics.

Characteristics	
Knees/patients	54/28
Ages (years)	32.6 ± 7.7
Male/female	13/15
Side (right/left)	28/26
Height (cm)	165.4 ± 8.2
Weight (kg)	61.1 ± 11.0
BMI (kg/m^2^)	22.1 ± 2.5
Follow-up (month)	25.6 ± 6.8

### Preoperative planning

The preoperative plan was completed by a senior orthopedic surgeon Qiao Feng. All patients were taken preoperatively with a standard anteroposterior full-length lower limb weight-bearing radiography.

We input the standard plain AP standing whole-leg radiographs into the SolidWorks 2016 (Dassault Systemes, USA) and corrected the radiograph magnification so that the software measured according to the scale marked on the radiograph.

**Figure 1:**
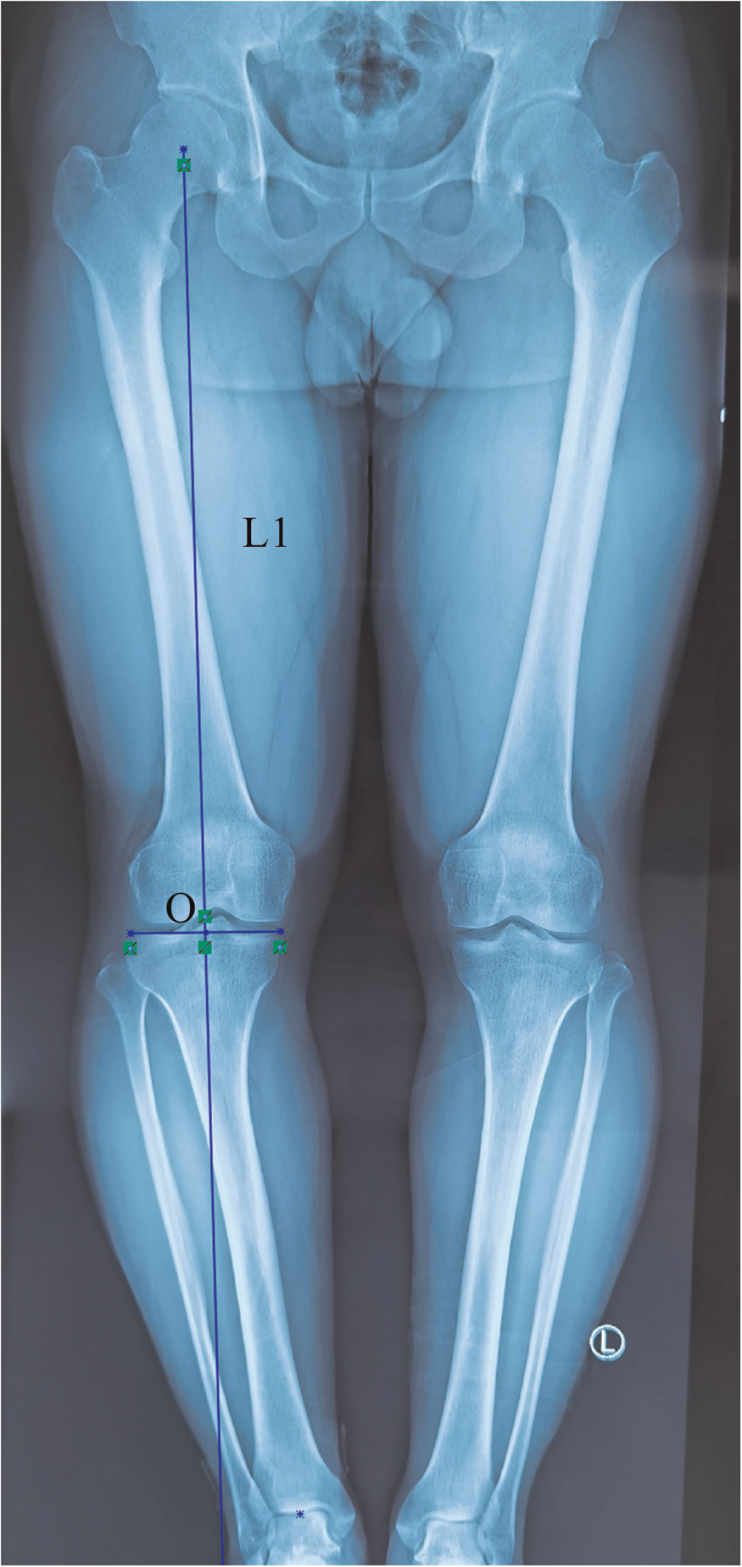
The target weight-bearing point (O) was set at 50% of the tibial plateau, and L1 represents the target weight-bearing line.

**Figure 2:**
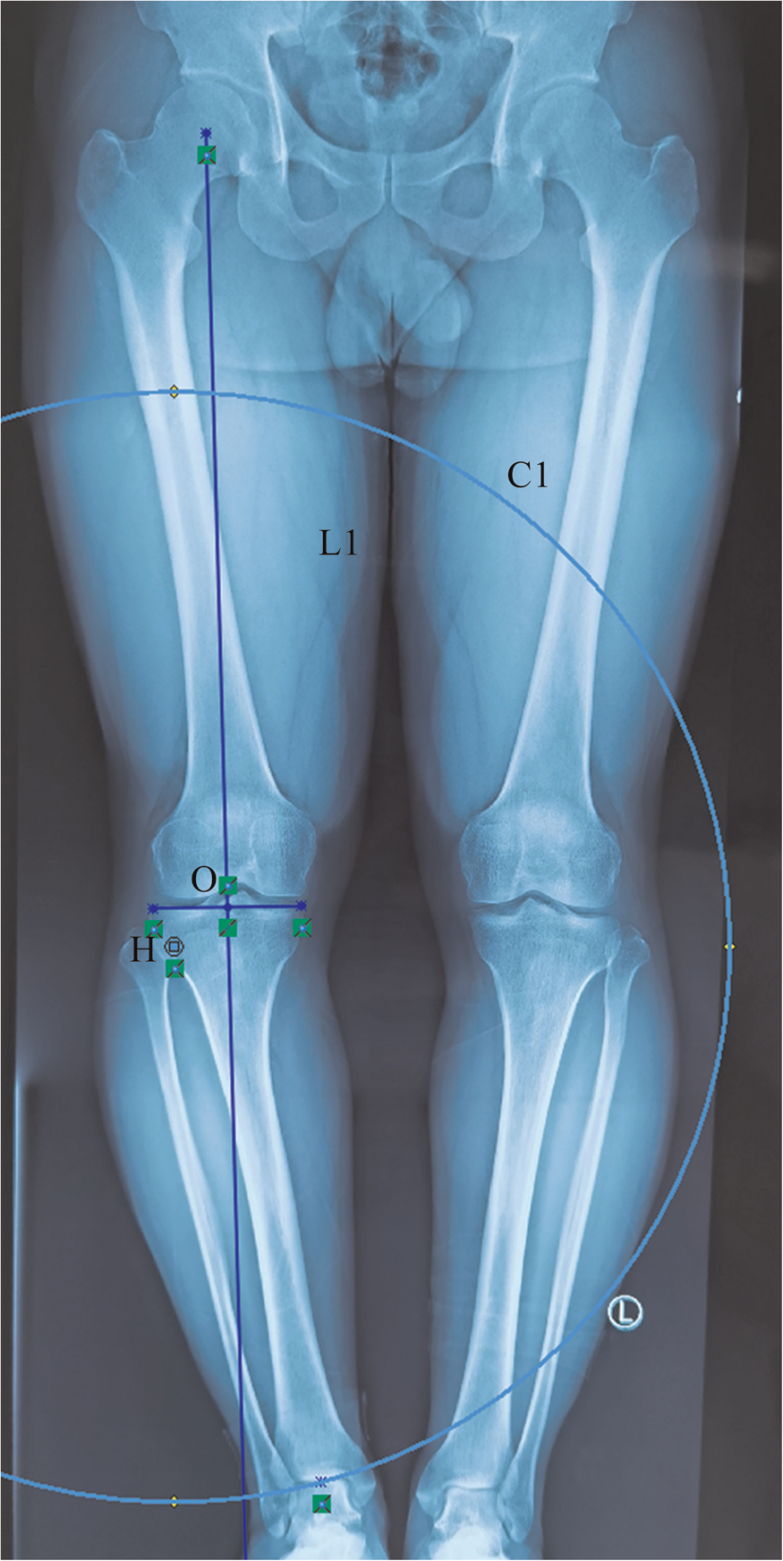
The site 15-mm distal to the lateral tibial plateau and 8-mm medial to the lateral cortex of the proximal tibia were selected as the hinge point (H). With point H as the center, a concentric circle was drawn through the center of the ankle joint (C1).

**Figure 3:**
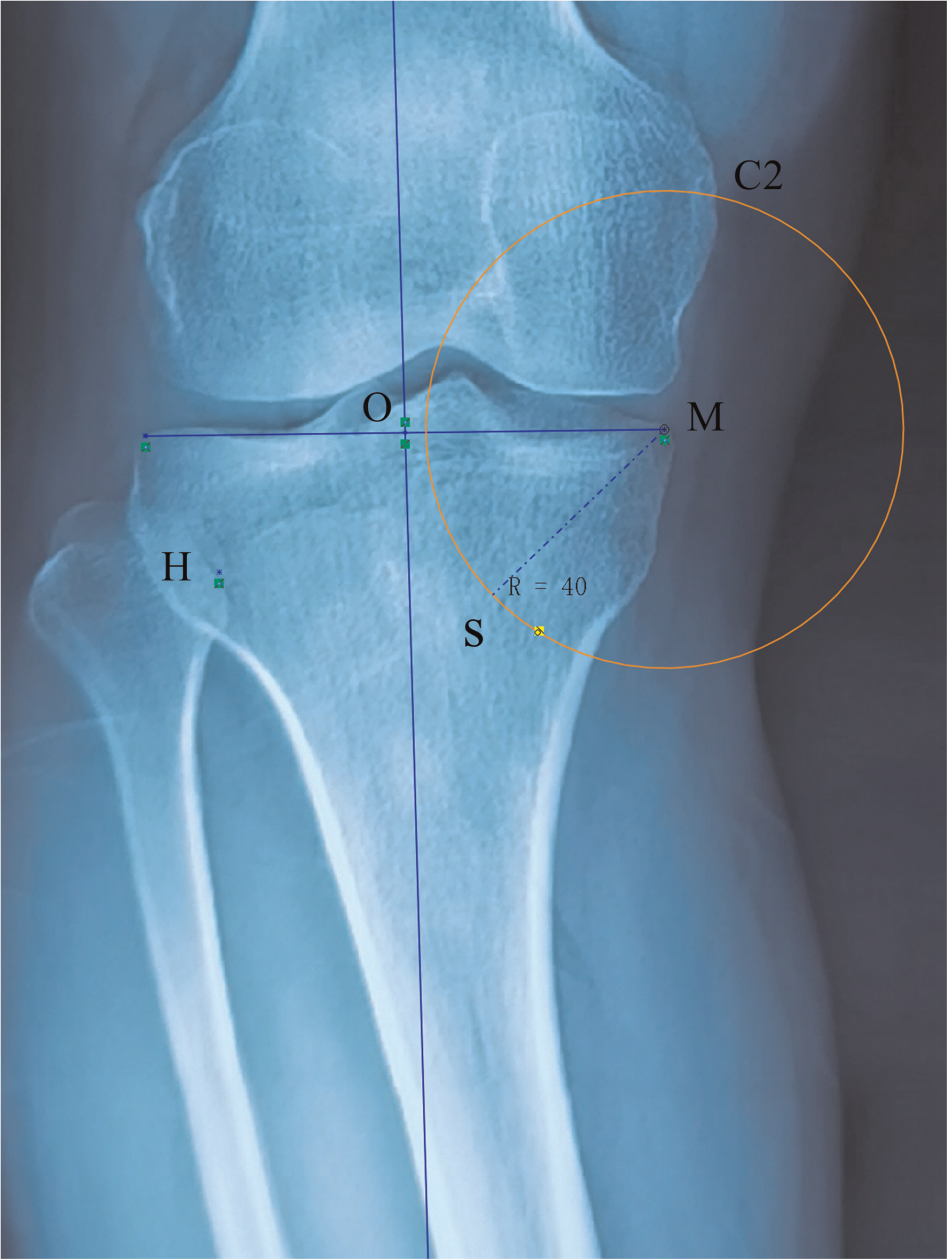
Point M represents the medial border of the tibial plateau. With M as the center and a concentric circle was drawn with a radius of 40 mm, which intersected at point S of the medial cortex of the tibia. Point S was selected as the osteotomy site.

**Figure 4:**
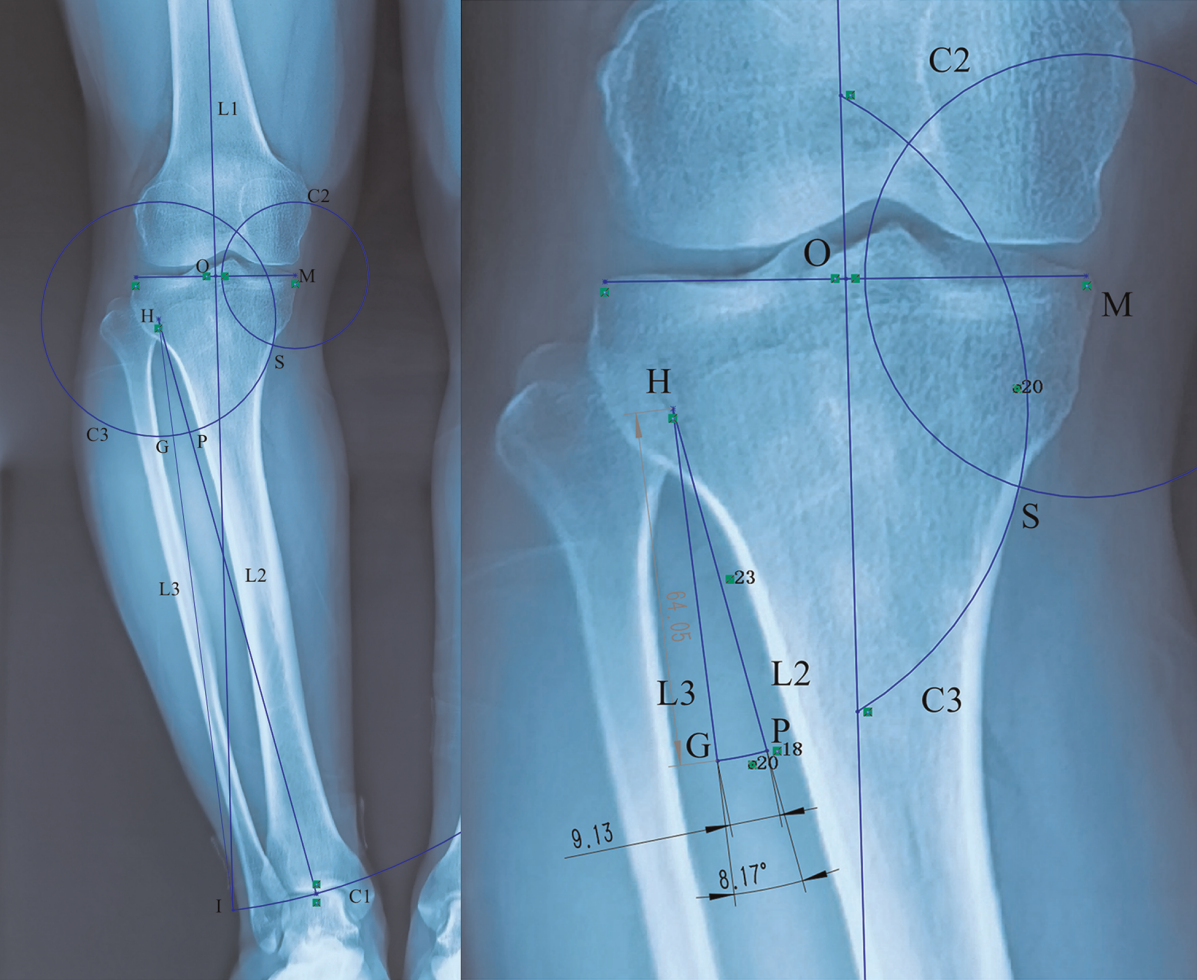
The H point was used as the center, and the distance from the H point to the ankle joint center (L2) was used as the radius to draw a concentric circle, L1 intersected at the I point, and line segment HI (L3) was drawn. The angle formed by L2 and L3 indicates the correction angle. Taking point H as the center, HS was used as the radius to draw a concentric circle that intersected L2 at point P and L3 at point G, while the length of the line segment PG indicated the length of the correction gap.

The following measurements were made:

1. Draw a line from the center of the femoral head to the midpoint of the tibial plateau and extend it distally beyond the ankle joint. We defined this line as the target weight-bearing line (Figure 1).
2. Mark the lateral hinge point. We selected a point 15-mm distal to the lateral tibial plateau and an 8-mm medial to the proximal lateral cortex of the tibia as the lateral hinge point. Make a concentric circle with a lateral hinge point as the center, passing through the center of the ankle joint and the target weight-bearing line (Figure 2).
3. The osteotomy site was marked. We took the medial edge of the tibial plateau as the center, with a radius of 40 mm for the concentric circles, and the intersection with the medial cortex of the tibia was marked as the osteotomy site (Figure 3).
4. A line was made connecting the lateral hinge point and the center of the ankle joint, with a line connecting the lateral hinge point and the intersection of the target weight-bearing line and the circle. The angle formed by the two lines served as the predicted correction angle.
5. Concentric circle was made with the lateral hinge point as the center and pass through the osteotomy site, and intersects the target WBL and the line from the lateral hinge point to the center of the ankle joint. The distance between the two intersection points was considered as the predicted correction gap (Figure 4).

### Surgical techniques

All surgeries were performed by the same senior orthopedic surgeon. The patient was asked to lay down in a supine position, the affected limb was routinely disinfected, and the tourniquet was applied. Create a longitudinal incision on the anterior medial side of the tibia with a length of 4 cm–6 cm. The skin was cut subcutaneously and the deep fascia sequentially. The 4-cm distal end of the medial tibial plateau was used as the osteotomy site (using a 4-cm long-cut Kirschner wire to determine under fluoroscopy), and a ф 2.0 Kirschner wire was inserted oriented medially to laterally, to the target hinge point (approximately 15-mm distal to the lateral tibial plateau, 8-mm inside the outer edge of the tibia). After fluoroscopy confirmed the correct position, the osteotomy line parallel to the tibial slope on the sagittal plane was marked. The pes anserinus tendons were dissected and the superficial medial collateral ligament was freed and retracted along the designed osteotomy line. Next, 2 Kirschner wires were inserted again on the marked osteotomy line, and 1 Kirschner wire was inserted into the posterior side of the tibial tubercle. After a blunt retractor was inserted posterior to the medial collateral ligament and the tibia to protect the neurovascular structures posterior to the incision line, an oscillating saw with 0.9-mm-thick saw blade was used to perform biplane osteotomy. During the sawing process, as per the preoperative plan, the sawing depth was controlled by the length scale of the saw blade. Then, stepwise insertion of 3–5 coupled chisels was performed into the osteotomy line and the spreader was finally used to gradually open out the medial cortex carefully, and then a trimmed tape was prepared during the operation, keeping the length exactly equal to the distance calculated preoperatively using the SolidWorks plus Saw blade thickness. When the opening reached the target distance, the laminar spreader was inserted into the posteromedial cortex of the tibia to maintain the realignment position. Finally, the TomoFix plate and locking screws (TomoFix, Synthes GmbH, Switzerland) were used to fix the osteotomized tibia. We did not use intraoperative fluoroscopy to examine the mechanical axis. The pes anserinus was not reconstructed when the wound was closed. If the tension of the medial collateral ligament was excessively large, the pie-crusting technique was used to loosen it until the tension was appropriate. None of the patients' lateral hinges were broken during the operation. We performed structural bone grafting for an opening distance >15 mm. On the first day after the operation, quadriceps and range-of-motion exercises were initiated. Full weight-bearing was allowed on the second day of surgery.

### Radiographic measures

**Figure 5:**
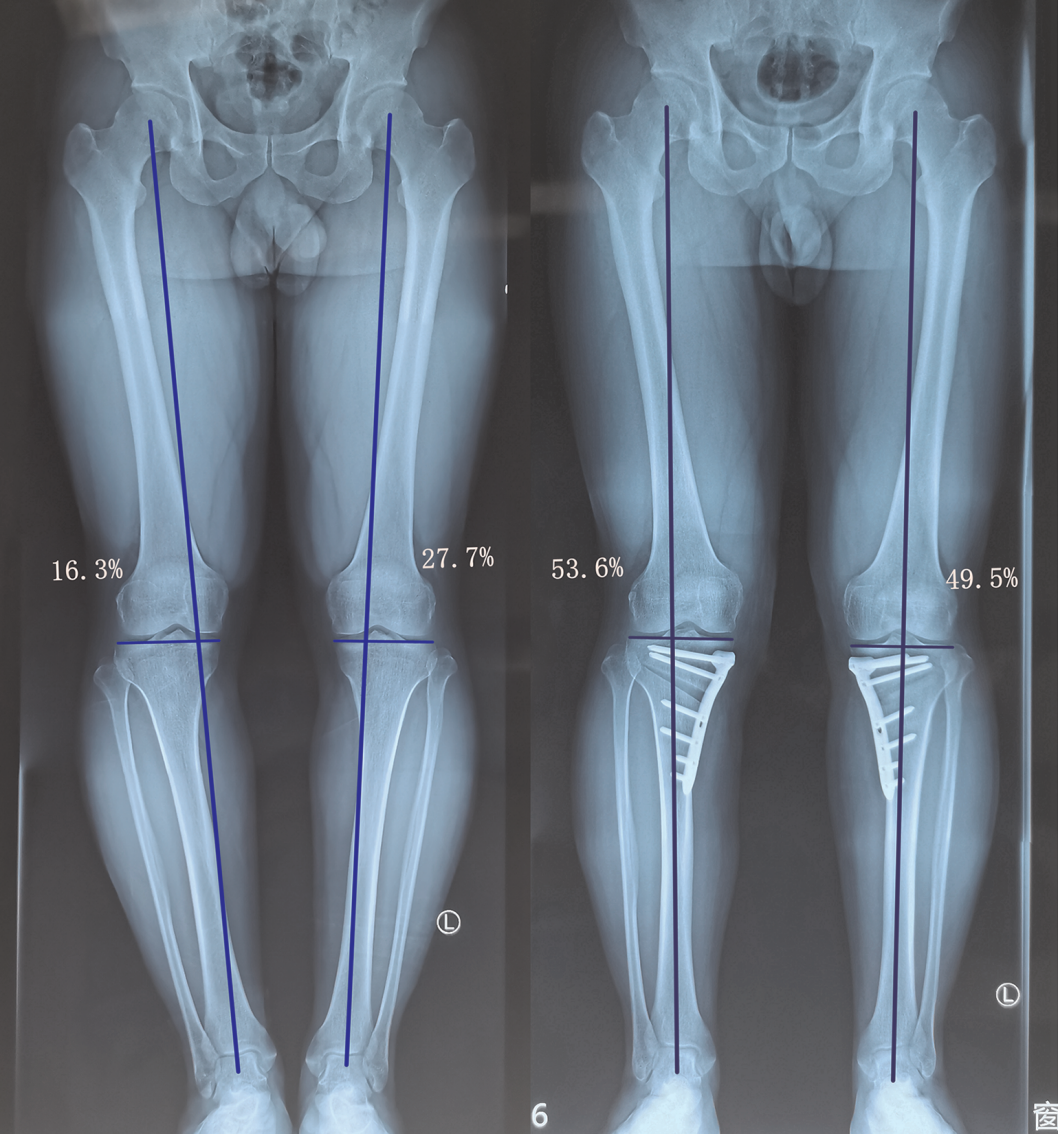
A 37-year-old man presented with medial compartment osteoarthritis (K-L grade I) and varus deformity in both knees. The WBL ratios of the left and right sides were 27.7% and 16.3% preoperatively, and 49.5% and 53.6% postoperatively, respectively.

All measurements were performed on the AP standing whole-leg radiographs using picture archiving and communication systems (PACS) (Synapse, Fujifilm Inc., Tokyo Japan) before surgery and at 6 -weeks postoperative follow-up. (1) The percentage of the WBL passing through the tibial plateau (calculated from the medial plateau) (Figure 5); the acceptable postoperative range was set to 50 ± 5% (range: 45%–55%), and percentages lower or higher than this range were defined as under- or over-correction, respectively. (2) Mechanical femorotibial angle (mFTA). (3) Mechanical medial proximal tibial angle (mMPTA). (4) The correction angle. (5) Preoperative correction distance. Due to the occlusion of the plate, the correction distance of the osteotomy site on the medial tibia cannot be measured after the operation.

All measurements were performed by 2 observers who did not participate in the operation. After 3 weeks, the measurement was performed again. The intraclass correlation coefficient (ICC) was applied to determine the reliability of the measurement. The ICC values were characterized as follows: poor agreement (<0.40), fair to good agreement (0.40–0.75), and excellent agreement beyond chance (>0.75). The measurement data used the mean value of the first measurement of the 2 observers.

### Clinical evaluation

Clinical outcome assessment used the knee social score (KSS) pre-operatively and at the final follow-up. The KSS comprises two parts: a knee score, which includes pain, stability, and a range of motion (ROM), and a function score, which includes the patient's ability to walk and climb stairs and the need for ambulatory aids.

### Statistical analysis

Statistical analysis was performed using PASW Statistics ver.18.0 (SPSS Inc., Chicago, IL, USA). All measurement data were expressed as mean ± standard deviation. The paired *t*-test was used to compare the preoperative and postoperative parameters. *P* < 0.05 was set to indicate a statistically significant difference.

## Results

The Kolmogorov–Smirnov test showed that all data followed the normal distribution pattern. ICC and interclass correlation coefficients for the reproducibility of all parameters were >80% (Table 2).

**Table 2:** Intraclass correlation coefficient of radiographic parameters.

	Intrarater agreement	Interrater agreement
Preop. WBL	0.97	0.95
Postop. WBL	0.91	0.95
Preop. MPTA	0.99	0.87
Postop. MPTA	0.87	0.83
Postop. wedge angle	0.95	0.89
Preop. mFTA	0.97	0.90
Postop. mFTA	0.96	0.89

The WBL ratio on the tibial plateau was corrected from the preoperative mean of 16.8% ± 13.0% (range, −37.3% to 36.7%) to the postoperative mean of 50.5% ± 4.4% (range, 40.6% to 62.3%) (*t* = 53, *p* < 0.001) (Table 3).

**Table 3:** Results of HTO using the SolidWorks methods.

		*T* value	*P* value
**Weight-bearing line (%)**			
Preoperative	16.8 ± 13.0	53	<0.001
Postoperative	50.5 ± 4.4		
**Wedge angle (°)**			
Preoperative	8.5 ± 3.5	−1.745	0.087
Postoperative	8.8 ± 2.9		
**Mechanical femur-tibia angle (°)**			
Preoperative	varus 6.4 ± 2.8	−18.605	<0.001
Postoperative	valgus 1.2 ± 1.3		
**Mechanical medial proximal tibial angle (°)**			
Preoperative	83.4 ± 2.7	−14.818	<0.001
Postoperative	89.3 ± 1.9		
**Knee Society knee score**			
Preoperative	76.4 ± 8.2	−18.843	<0.001
Postoperative	95.0 ± 3.6		
**Knee Society function score**			
Preoperative	80.7 ± 9.1	−15.165	<0.001
Postoperative	95.7 ± 4.9		

Forty-three knees of correction were found to be within the acceptable range (79.6%, 49.3% ± 2.6%), 2 knees were under-corrected (3.7%, 41.8% ± 1.7%), and 9 knees were over-corrected (16.7%, 58.1% ± 1.8%) based on the WBL on the tibial plateau.

The mFTA was varus 6.4 ± 2.8° before the surgery and valgus 1.2 ± 1.3° after the surgery (Table 3). The preoperatively planned opening angle was 8.5 ± 3.5°, and the postoperative measurement correction angle was 8.8 ± 2.9°. No statistical difference was observed in the preoperative opening angle and postoperative correction angle (*t* = 1.745, *P* = 0.087) (Table 3). The average planned preoperative opening gap was 8.9 ± 3.7 mm. The KSS was significantly improved after the surgery (95.0 ± 3.6) compared with the preoperative KSS (76.4 ± 8.2). The functional score was improved after the surgery (95.7 ± 4.9) compared with the preoperative functional score (80.7 ± 9.1) (Table 3).

## Discussion

Digital preoperative planning has become the mainstream in OWHTO. He et al. (14) used the OsteoMaster software for preoperative planning, and the operation time and the number of x-ray fluoroscopy were significantly reduced compared with the traditional Miniaci method. The accuracy, depth, open height, correction angle, FTA, and WBL ratio of osteotomy were not reduced compared with the traditional Miniaci method. Kim et al. (11) used PACS technology for preoperative planning of open-wedge HTO and used the Miniaci method to measure the preoperative tibial plateau WBL ratio, correcting angle, and opening distance and to compare them with those of the last postoperative follow-up. No statistical difference was found between parameters obtained by preoperative planning and postoperative x-ray radiography, indicating that PACS technology can be used for HTO preoperative planning.

Lee et al. (19) used the PACS-photoshop method and the Real-size paper template method for OWHTO preoperative planning, compared the two methods prospectively, and found that the former is highly reliable. Later, they (20) reviewed 72 cases treated by open-wedge HTO using the PACS-Photoshop method for preoperative planning. The postoperative measurement average correction gap was 10.8 mm; the correction gap of <10.8 mm was divided into one group, and that higher than 10.8 mm was divided into another group. By comparing the postoperative correction gap with the measured preoperative correction gap, they found that when the correction gap is large, the difference between the postoperative and preoperative WBL ratios increases. However, it did not deviate toward the side of either over-correction or under-correction. Schröter et al. (16) studied the inter-group reliability of the digital software PreOPlan and mediCAD for open-wedge HTO preoperative planning and found that both the software preoperative plans have a high degree of inter-group reliability, and are unaffected by the experience of the measurer. They (15) used the mediCAD digital software for OWHTO preoperative planning and closed distal femur osteotomy (DFO) preoperative planning for severe knee varus osteoarthritis, and good imaging and clinical results were obtained.

Some studies recently reported the use of 3D-planned patient-specific instrumentation (PSI) (21) and navigation system (22) to perform open-wedge HTO and a more accurate WBL ratio, especially tibial slope was obtained. However, Tardy et al. (23) performed a multi-center non-randomized controlled prospective observational study by comparing the parameters of 126 patients in the navigation group, PSI group, and traditional group using the Miniaci method in 11 centers. The results showed that none of the 3 techniques were superior in achieving target correction at 1 year. All the 3 techniques were reliable and precise in HTO planning.

The SolidWorks software is a powerful engineering drawing software. We have developed its imaging measurement function in the medical field. SolidWorks can mark equidistant points by making concentric circles. When SolidWorks measures the length, the accuracy can reach 0.0000001 mm and when it measures the angle, the accuracy can reach 0.0001°. However, the accuracy of PACS measuring length is only 0.01 mm, while the accuracy of measuring angle length is 1°. SolidWorks uses the principle that all radii of concentric circles are equal in length to reduce the measurement steps and the measurement errors. Compared with PACS, SolidWorks not only reduced the errors and improved the accuracy of the measurement but also reduced the measurement steps and improved the efficiency of the measurement. When compared to other 2D digital measurement software, SolidWorks measurement accuracy is the highest.

When open-wedge HTO is used to treat medial knee osteoarthritis, the optimal position of the lower limb WBL on the tibial plateau is debatable. Most studies (4, 5, 11, 14, 18, 22) have used the Fujisawa point as the target load line passing point of the tibial plateau. We used the Fujisawa point as the target point for performing open-wedge HTO in patients aged >45 years and having K-L grade II or higher for medial knee osteoarthritis. However, young and active patients <45 years of age and K-L grade did not exceed grade I for medial knee osteoarthritis when 50% of the tibial plateau was the target point. The short-term results of all cases were desirable but the long-term results will need follow-up.

Yoon et al. (5) compared the use of PACS for preoperative planning and intraoperative use of a cable method for open-wedge HTO using the tibial plateau target WBL ratio ±5° as the acceptable range of correction and found that the acceptable range of the intraoperative wire method was 55%, whereas that of the PACS method was 71.8%. Miniaci et al. (24) reported that only 50% of their cases were within the acceptable range (±10%) of correction after proximal tibial osteotomy. Kim et al. (11) used the PACS method for preoperative planning of OWHTO, and the acceptable range of correction (±5%) after surgery was 70%, under-correction was 20%, and over-correction was 10%. Using the SolidWorks software, we achieved the acceptable WBL ratio (±5%) of 79.6%, the under-correction of 3.7%, and the over-correction of 16.7%. The advantage of using the SolidWorks method is that fluoroscopy is not required to determine the alignment of the lower extremities during the surgery, thus reducing the x-ray radiation damage and the surgical time.

This study has several limitations. First, this study is a retrospective study having a small sample size and short follow-up time. Further studies using a large sample size and more patients are required to confirm the study findings. It would be best to perform a prospective randomized controlled study with a longer follow-up time to obtain more reliable results. Second, Sabharwal et al. (25) reported that the standing full-length anteroposterior radiograph magnification rate of the lower limbs was 4.6%. Their minimum patient-to-tube distance was 203 cm, whereas the minimum distance in our image center was 180 cm. Therefore, our magnification may be greater, which may be the reason for obtaining overall large values including the WBL ratio. Third, during the surgery, we controlled the posterior slope of the tibia by making the sagittal plane osteotomy line parallel to the tibial joint line and ensuring that the two osteotomy planes of the tibial tubercle were parallel. However, we did not compare the posterior tibial slope before and after the surgery. Finally, we kept the WBL in the center of the tibial plateau, which is debatable.

## Conclusion

The SolidWorks software showed high accuracy and reliability in preoperative planning of open-wedge HTO in patients with medial compartment knee arthritis.

## Data Availability

The raw data supporting the conclusions of this article will be made available by the authors, without undue reservation.
